# Redescription of the red-striped soft scale, *Pulvinaria tenuivalvata* (Newstead), with a new synonymy (Hemiptera, Coccomorpha, Coccidae)

**DOI:** 10.3897/zookeys.647.11225

**Published:** 2017-01-25

**Authors:** Soad I. Abdel-Razak, Daniele Matile-Ferrero, Adeline Soulier-Perkins

**Affiliations:** 1 Agricultural Research Centre, Plant Protection Research Institute, Scale Insects and Mealybugs Dept., Alexandria, Egypt; 2 current: University of Hafr Albatin, Faculty of Sciences, Biology Dept., Saudi Arabia; 3 Muséum national d’Histoire naturelle, Institut de Systématique, Evolution, Biodiversité, ISYEB-UMR 7205 MNHN-CNRS-UPMC-EPHE, 57 rue Cuvier, CP 50, FR-75005 Paris, France; 4 Muséum national d’Histoire naturelle, Mécanismes adaptatifs et Evolution, MECADEV-UMR 7179, MNHN-CNRS, 57 rue Cuvier, CP 50, FR-75005 Paris, France

**Keywords:** Coccidae, Egypt, pest, sugarcane, synonymy

## Abstract

The soft scale, *Pulvinaria tenuivalvata* (Newstead, 1911), is a major pest of sugarcane in Egypt. This paper provides a redescription and illustration of the adult female based on a microscopic study of the morphology of several adult female specimens and of the type series illustrated by De Lotto (1965) on citronella grass from Uganda. Two paratypes of *Pulvinaria saccharia* De Lotto, 1964 are also studied and the name is placed here as a junior synonym of *Pulvinaria tenuivalvata*.

## Introduction

Sugarcane, *Saccharum officinarum* L. (Poaceae) is one of the main crops in Egypt, and the control of pests on this crop is very important. The red-striped soft scale *Pulvinaria tenuivalvata* is a major pest since 1992, when it was observed for the first time and named as *Pulvinaria elongata* Newstead (Karam and Abou-Alkhair 1992). It attacks leaves, causing a major reduction in crop yield due to depletion of sap, production of honeydew and growth of sooty mould. Early and heavy infestations have resulted in complete yield lost (El-Serwy et al. 2008).

The species was described by Newstead (1911) as *Lecanium tenuivalvatum*, based on adult females, all of which were heavily parasitisied, infesting citronella grass (*Cymbopogon citratus*) in Uganda. De Lotto (1964) described and illustrated for the first time *Pulvinaria saccharia* collected on leaves of sugar cane in Durban, Natal, and stated that the species is structurally very closely related to *Pulvinaria tenuivalvata* (Newstead, 1911). The same author (De Lotto 1965) redescribed and illustrated the adult female of *Pulvinaria tenuivalvata* from a single specimen from the type locality and the type host plant. Williams (1982), in his study of *Pulvinaria iceryi* (Signoret) and its allies on sugarcane and other grasses, separated *Pulvinaria tenuivalvata* from five *Pulvinaria* species, giving a key and commenting on the great similarity of these five species, *Pulvinaria elongata* Newstead, *Pulvinaria iceryi* (Signoret), *Pulvinaria saccharia* De Lotto, *Pulvinaria sorghicola* De Lotto and *Pulvinaria tenuivalvata* (Newstead). He stated that *Pulvinaria tenuivalvata* is very close to *Pulvinaria saccharia*. Watson and Foldi (2002) discussed the identity of the pest on sugarcane in Egypt and identified the species as *Pulvinaria tenuivalvata* (Newstead), although it had previously been identified as *Pulvinaria elongata* Newstead (Karam and Abu-Elkhair 1992) and *Saccharolecanium krugeri* (Zehntner) (Ali et al. 1997). In 2001, Ghabbour and Hodgson described and illustrated the 1^st^ instar nymph and 2^nd^ and 3^rd^ instar female nymphs of *Pulvinaria tenuivalvata* and provided a key.

The present paper redescribes and illustrates the adult female of *Pulvinaria tenuivalvata* (Newstead) in detail. In addition, we were able to study two paratypes of *Pulvinaria saccharia* De Lotto, 1964 and conclude that the name *Pulvinaria saccharia* is a junior synonym of the name *Pulvinaria tenuivalvata*.

## Materials and methods

Slide-mounted adult females were studied from the entomology collections at The Natural History Museum, London, U.K. (BMNH) and Muséum national d’Histoire naturelle, Paris, France (MNHN). The photos were produced using with a Leica DFC 420 camera and the software Leica Application Suite, version 2.8.1. The drawings were made using the software Illustrator CS6 version 16.0.0. Morphological terms follow those by Hodgson (1994) and Qin and Gullan (1992).

## Taxonomy

### Pulvinaria tenuivalvata

Taxon classificationAnimaliaHemipteraCoccidae

Newstead, 1911

Lecanium tenuivalvatum Newstead, 1911: 92.
Pulvinaria tenuivalvata (Newstead), De Lotto 1965: 217.
Pulvinaria elongata Newstead; Karam and Abu-Elkhair 1992: 587, misidentification.
Saccharolecanium krugeri (Zehntner); Ali et al. 1997: 149, misidentification.
Pulvinaria saccharia De Lotto, 1964: 863, 2 paratype adult females, South Africa, Natal, Durban, on Saccharum officinarum, J. Munting, 25/03/1964 (BMNH); De Lotto 1966: 468; Hodgson 1968: 207; 1969: 29, 30; Qin and Gullan 1992: 121. **syn. n.**

#### Description of the adult female.

**Figure 1.**
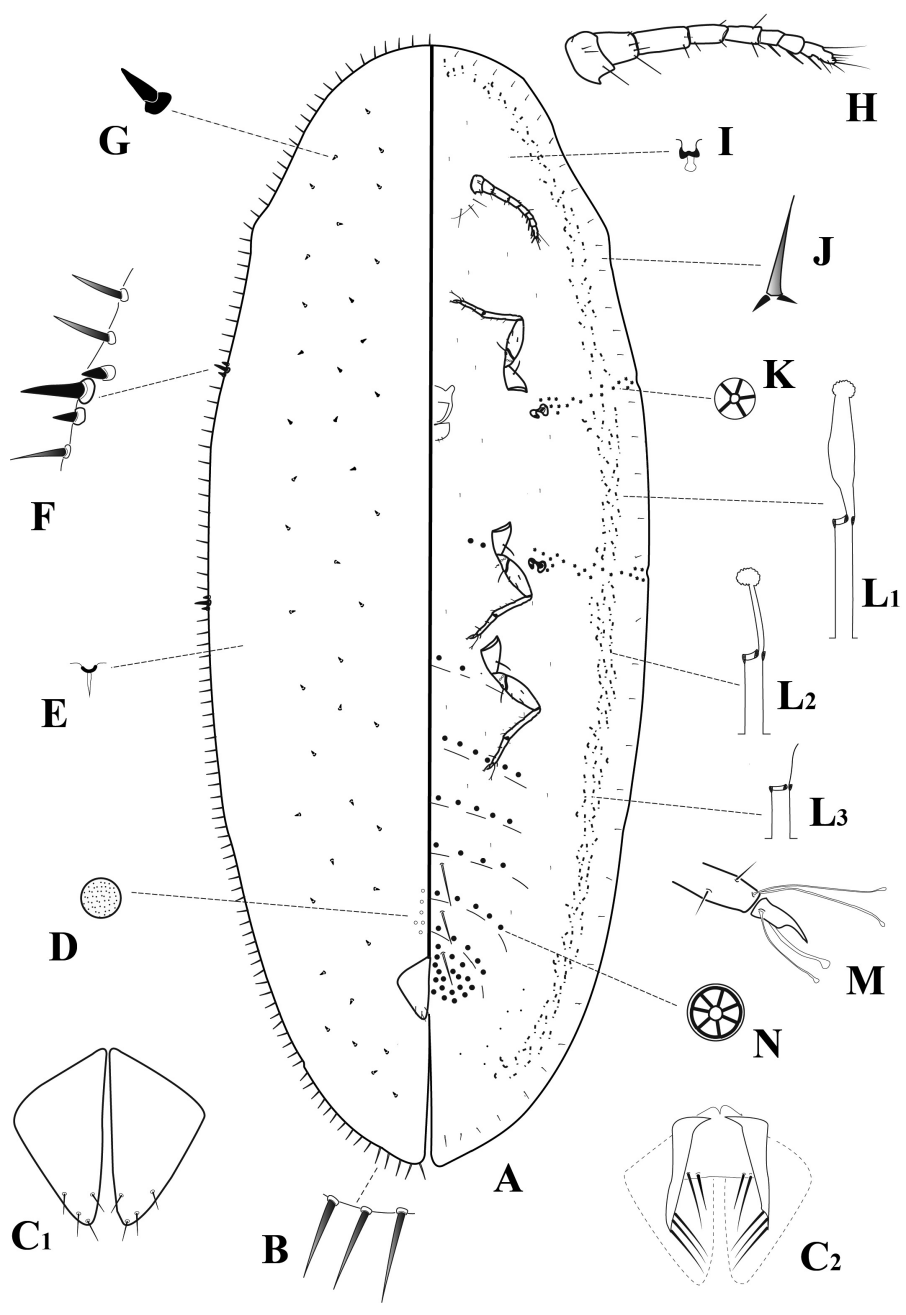
*Pulvinaria tenuivalvata* (Newstead). **A** Body, venter and dorsum **B** marginal setae **C1** anal plates, dorsal view **C2** ano-genital fold **D** dorsal discoidal pore **E** dorsal filamentous pore **F** spiracular setae **G** dorsal seta **H** antenna **I** ventral microduct **J** ventral submarginal seta **K** spiracular disc-pore **L** ventral tubular ducts of three types: L1, type I, L2, type II, L3, type III **M** claw digitules unequal **N** multilocular disc-pore.

**Figures 2–6.**
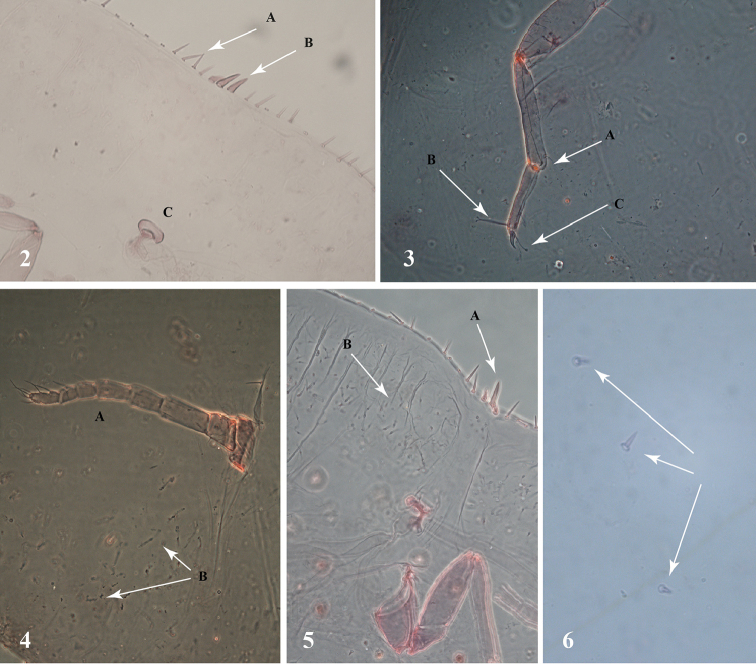
*Pulvinaria tenuivalvata* (Newstead). **2A** marginal setae **2B** anterior spiracular setae **2C** anterior spiracle **3A** tibio-tarsal articulatory sclerosis **3B** tarsal digitules **3**, claw digitules unequal **4A** antenna **4B** ventral tubular ducts of the type I **5A** posterior spiracular setae **5B** ventral tubular ducts of the type I **6** dorsal conical setae.

Figs 1–6. The adult female of *Pulvinaria tenuivalvata* is very elongate, convex with the cephalic region flattened. The body colour varies from pale crimson to flesh-coloured with two irregular longitudinal bands of bright crimson on the dorsum. No true ovisac is formed, except under the body where it extends forward to the eyes and may project slightly from beneath the female body.

*Body* (Fig. 1A): very elongate, oval, narrow at both ends, 3.4–6.5 mm long, 1.5–2.5 mm wide. Derm membranous. Anal cleft rather shallow ranged from 0.70 to 0.74 mm in length. Stigmatic clefts poorly developed.

*Margin*: marginal setae (Fig. 1B) numerous, slender and pointed, with well-developed basal-sockets, distributed in one row with 12-18 setae on each side between the anterior and posterior stigmatic clefts, mostly about 35–40 µm long, a few only approximately 20 µm long, the longest setae similar in length to the median stigmatic setae (Fig. 2A). Three stigmatic setae present (Fig. 1F) in each stigmatic cleft; these setae short, stout, pointed, the median seta longest (Fig. 2B), straight or more-or-less curved, variable in size and thickness, 25–40 µm long, lateral spiracular setae also variable in size and shape, each 15–20 µm long, pointed (Figs 2B, 5A).

*Dorsum*: dorsal setae (Fig. 1G) stout, conical, 10–15 µm long, not lanceolate (Fig. 6), scattered all over body surface. Submarginal tubercles absent. Preopercular pores minute (Fig. 1D) about 3 µm in diameter, grouped in small number (6–18) anteriorly to the anal plates. Filamentous pores minute (Fig. 1E), evenly distributed. Anal plates (Fig. 1C1) together quadrate, each plate 125–140 µm long and 70–75 µm wide. Each plate with four short setae; one apical, one subapical, one inner margin seta and one outer margin seta. Ano-genital fold (Fig. 1C_2_ with two pairs of long anterior marginal setae (a median pair each nearly 45 µm long and a submedian pair each around 65 µm long) and three pairs of long lateral margin setae, 55–65 µm. Anal ring with four pairs of long setae and two rows of pores.

*Venter*: submarginal setae (Fig. 1J) setose, approx. 7 µm long, placed in a submarginal row. Interantennal setae long, present in three pairs. Prevulvar setae long, one pair on each of the three prevulvar segments. Minute ventral setae evenly distributed. Antennae well developed, eight segmented (Figs 1H, 4), 300–370 µm long, 3^rd^ segment longest. Legs well developed, each with a distinct tibio-tarsal articulatory sclerosis (Fig. 3A), claw without distinct denticle (Fig. 3C). Tarsal digitules, slender, knobbed at apex (Figs 1M, 3B), 45–50 µm long. Hind trochanter + femur 200–260 µm long. Claw digitules unequal, one twice diameter of other, both slender, of the same length, each slightly knobbed at the apex (Figs 1M, 3C), 35–40 µm long. Anterior and posterior spiracles well-developed. Spiracular disc-pores (Fig. 1K) with five loculi, some with four or three loculi, 3–4 µm in diameter, present in a narrow band extending from each spiracle to margin. Multilocular disc-pores (Fig. 1N) with 7–8 loculi, some pores occasionally with fewer loculi, approx. 5 µm in diameter, numerous around vulva, in single rows on all preceding abdominal segments and a few present, submedially, on metathorax and mesothorax. Ventral microducts present, minute (Fig. 1I), sparse. Ventral tubular ducts (Fig. 1L) in a submarginal band, 4 - 6 ducts wide, numerous around entire body, except caudal area and head region where they are sparse. Three types of ventral tubular ducts are present, all of similar diameter. Type I (Fig. 1L1) long and narrow, numerous, with outer ductule about 20 µm long, approx. 3 µm in diameter, inner ductule approx. 24 µm long, longer and wider than outer ductule and with a large terminal gland. Type II (Fig. 1L2) shorter than type I, less numerous, 10 µm long and 3 µm in diameter, inner ductule 7 µm long, narrower than outer one and with a terminal gland. Type III (Fig. 1L3), the shortest, with outer ductule 5 µm long and inner ductule slender and short, without a terminal gland, very few in number.

#### Material examined.

**Egypt**: 100 km south of Cairo, Benisueif, on sugarcane and rarely on maize, M.A. Shalaby, ? 1997 (BMNH); upper Egypt, Giza, on sugarcane leaves, 1997 (BMNH); Qena Governorate, Luxor and Qus (700 km south of Cairo), on undersides of sugarcane leaves, S.A. El-Serwy, 01/1999 (BMNH); Giza region, 40-80 km south of Cairo, on *Saccharum officinarum* (commercial), S.A. El-Serwy, 10/08/1999 (BMNH); upper Egypt, Qena Governorate, on sugarcane leaves, 12/2000 (BMNH); on sugarcane, S. Ramadan, 2011 (MNHN). **Uganda**: Entebbe, on citronella grass, C.C. Gowdey, 18/02/1910, G. De Lotto, 1960, B.M. 1963-473 (BMNH). **South Africa**, Natal, Durban, on *Saccharum officinarum*, J. Munting, 25/03/1964, *Pulvinaria saccharia*, 2 paratypes, B.M. 1964-662 (BMNH).

#### Host plants.

The main host plant in Egypt is sugarcane, *Saccharum officinarum*, but it has also been recorded from several other Poaceae in Egypt: *Imperata cylindrica*, *Sorghum vulgare saccharatum*, and *Zea mays*. The species is known on *Cymbopogon citratus* (citronella grass) and *Pennisetum purpureum* in Uganda (García Morales et al. 2016), and from Zimbabwe and South Africa (Natal), on *Saccharum officinarum* (as *Pulvinaria saccharia*).

#### Comments.

Two paratypes of *Pulvinaria saccharia* De Lotto, 1964, have been examined, both adult females. The dorsal setae are short, strong and spiniform, but certainly not lanceolate as stated by the previous authors (De Lotto 1964; Williams 1982). The claw digitules are unequal, one much thicker than the other one, but of the same length. This character was first observed by De Lotto (1964; 1965) and confirmed by Williams (1982) and Watson and Foldi (2002). These two paratypes show the presence of three types of ventral submarginal tubular ducts, as always. *Pulvinaria saccharia* has ventral multilocular disc-pores on the metathorax and the mesothorax. The range of setae between the anterior and the posterior spiracles is about 29-31. The combined length of hind trochanter plus femur is about 200–220 µm. On the appearance in life of *Pulvinaria saccharia*, De Lotto (1964) mentioned that “*Pulvinaria saccharia does not form any ovisac but a thin layer of white cottony wax laid beneath the body along the margin*”. A similar type of ovisac on adult females of *Pulvinaria tenuivalvata* was observed in Egypt. All these characters fall within the range of the morphological characters of *Pulvinaria tenuivalvata*, so *Pulvinaria saccharia* is here treated as a synonym of *Pulvinaria tenuivalvata*.

## Supplementary Material

XML Treatment for Pulvinaria tenuivalvata

## References

[B1] Ali MAM Farrag MM Shalaby SI (1997) First record of the sugar-cane scale *Saccharolecanium krugeri* (Zehntner) in Giza, Egypt. Bulletin of the Entomological Society of Egypt 75: 149–152.

[B2] De Lotto G (1964) A new species of *Pulvinaria* (Homopt.: Coccidae) attacking sugar cane in South Africa. South African Journal of Agricultural Science 7: 863–866.

[B3] De Lotto G (1965) On some Coccidae (Homoptera), chiefly from Africa. Bulletin of the British Museum of Natural History, Entomology 16: 175–239. https://doi.org/10.5962/bhl.part.21865

[B4] De Lotto G (1966) Another new species of *Pulvinaria* (Hom.: Coccidae) from sugar cane. South African Journal of Agricultural Science 9: 467–472.

[B5] El-Serwy A Guerrieri E Evans GA (2008) The parasitoid complex of the red-striped soft scale, *Pulvinaria tenuivalvata* (Newstead) (Hemiptera: Coccidae) and its long-term effect on the scale on sugarcane in Egypt. Proceedings of the XI International Symposium on Scale Insect Studies, Oeiras, Portugal, 24-27 September 2007 ISA Press Lisbon, 217–227.

[B6] García Morales M Denno BD Miller DR Miller GL Ben-Dov Y Hardy NB (2016) ScaleNet: A Literature-based model of scale insect biology and systematics. http://scalenet.info [accessed: 17 February 2016] 10.1093/database/bav118 PMC4747323 26861659

[B7] Ghabbour MW Hodgson CJ (2001) The immature stages of *Pulvinaria tenuivalvata* (Newstead) (Hemiptera: Coccidae). Bolletino di Zoologia Agraria e di Bachicoltura 33(3): 43–51.

[B8] Hodgson CJ (1968) Some *Pulvinaria* species (Homoptera: Coccidae) of the Ethiopian region. Journal of the Entomological Society of South Africa 30: 198–211.

[B9] Hodgson CJ (1969) Notes on Rhodesian Coccidae (Homoptera: Coccoidea): part III. Arnoldia 4(4): 1–42.

[B10] Hodgson CJ (1994) The Scale Insect Family Coccidae: An Identification Manual to Genera. CAB International, Wallingford, Oxon, 639 pp.

[B11] Karam H Abu-Elkhair S (1992) First record of *Pulvinaria elongata* Newstead (Homoptera: Coccidae) in Egypt. Alexandria Journal of Agricultural Research 37(1): 587–594.

[B12] Newstead R (1911) Observations on African scale insects (Coccidae). (No. 3). Bulletin of Entomological Research 2: 85–104. https://doi.org/10.1017/S0007485300001255

[B13] Qin TK Gullan PJ (1992) A revision of the Australian pulvinariine soft scales (Insecta: Hemiptera: Coccidae). Journal of Natural History 26: 03–164.

[B14] Watson G Foldi I (2002) The identity of the red-striped soft scale on sugarcane in Egypt, *Pulvinaria tenuivalvata* (Newstead) (Hemiptera: Coccidae). Bulletin of the Entomological Society of Egypt 79: 37–42.

[B15] Williams DJ (1982) *Pulvinaria iceryi* (Signoret) (Hemiptera: Coccidea) and its allies on sugar-cane and other grasses. Bulletin of Entomological Research 72: 111–117. https://doi.org/10.1017/S000748530005032X

